# The Selectivity of CK2 Inhibitor Quinalizarin: A Reevaluation

**DOI:** 10.1155/2015/734127

**Published:** 2015-10-19

**Authors:** Giorgio Cozza, Andrea Venerando, Stefania Sarno, Lorenzo A. Pinna

**Affiliations:** Department of Biomedical Sciences, University of Padova and CNR Institute of Neurosciences, Via Ugo Bassi 58B, 35131 Padova, Italy

## Abstract

Many polyphenolic compounds have been reported to inhibit protein kinases, with special reference to CK2, a pleiotropic serine/threonine kinase, implicated in neoplasia, neurodegenerative disease, and viral infections. In general however these compounds are not endowed with stringent selectivity. Among them quinalizarin (1,2,5,8-tetrahydroxyanthraquinone) turned out to be particularly potent (Ki = 0.058 *μ*M) and quite selective as judged by profiling it on a small panel of 70 protein kinases. Here, by profiling quinalizarin on a larger panel of 140 kinases we reach the conclusion that quinalizarin is one of the most selective inhibitors of CK2, superior to the first-in-class CK2 inhibitor, CX-4945, now in clinical trials for the treatment of cancer. Moreover here we show that quinalizarin is able to discriminate between the isolated CK2 catalytic subunit (CK2*α*) and CK2 holoenzyme (CK2*α*_2_ *β*_2_), consistent with *in silico* and *in vitro* analyses.

## 1. Introduction

Quinalizarin (1,2,5,8-tetrahydroxyanthraquinone) is a polyphenolic compound originally used in the manufacture of dyes and pigments. It has been considered a pollutant in waste waters from many textile industries since it is nonbiodegradable and very toxic to aquatic organisms. Quinalizarin is one of many tetrahydroxyanthraquinone isomers, presenting an asymmetric chemical structure responsible for peculiar chemical properties. It works as an acid-base indicator being orange in neutral/acidic solution, blue in mild base, and purple in strong base, thus presenting the deprotonation of one or two hydroxyl groups, respectively [1]. Its colorimetric properties have been exploited for determination of different metal ions concentrations thanks to its ability to form colored chelates. Many examples of this application have been reported since the early 1950s, for the detection of boron [2], uranium, molybdenum [3], and aluminium [4]. More recently, a spectrophotometric method, based on quinalizarin complexation reaction, has been applied to manganese and thallium estimation in water and biological samples [5, 6]. A similar method has also been performed to obtain the determination of two antiepileptics (gabapentin and pregabalin) in pharmaceutical formulations [7].

On the other hand, quinalizarin has been exploited in cancer research, being effective in different types of tumor cells (breast cancer [8], prostate cancer [9], and leukemia T cells [10]) and angiogenesis [11]. It has been suggested as a promising drug prototype against human ganciclovir-sensitive and ganciclovir-resistant cytomegalovirus [12] and reported to inhibit growth of HIV on human peripheral blood mononuclear cells [13, 14]. In 2009 quinalizarin has been identified as a potent and selective inhibitor of protein kinase CK2 through a computer aided virtual screening and biochemical evaluation [10] and demonstrated to be a cell permeable compound able to inhibit endogenous CK2 in HEK-293 and Jurkat cells at a concentration <5 *μ*M [10]. Protein kinase CK2 is a Ser/Thr enzyme composed of two catalytic (*α* or *α*′) and two regulatory (*β*) subunits, which phosphorylates an extraordinary number of substrates, at sites fulfilling S/T-X-X-E/D/pS/pY consensus [15]. CK2 is involved in many cellular processes, such as gene expression, differentiation, protein synthesis, and proliferation, but it is especially considered a global antiapoptotic agent [16–18]. It regulates the cell death/survival ratio, thus being implicated in many hallmarks of cancer such as angiogenesis and drug resistance and it is also overexpressed in cancer cells [17–22] which are addicted to its activity [23]. Moreover, important role of CK2 has been demonstrated in neurodegenerative diseases and virus/parasites proliferation [17, 24–27]. Given these premises, it is not surprising that quinalizarin is effective in many disease models in which CK2 is effectively implicated, thus confirming CK2 as the principal target of this molecule. Recently quinalizarin has provided a strong argument to support the concept that CK2 may represent an appealing target for prosenescence antitumor strategies [28]. From a molecular point of view a detailed crystallographic study of the binding mode between quinalizarin and CK2*α* subunit has been performed; initially cocrystallyzed with* Zea Mays* CK2 at pH 7.5 (PDB code: 3FL5 [10]), later the complex between quinalizarin and human CK2 was solved at pH 6.5 and 8.5 (PDB codes: 3Q9Z and 3Q9Y, resp. [29]).

Quinalizarin has been demonstrated to be an effective tool in research; it has promoted the identification of CK2 roles in the regulation of the insulin production on pancreatic *β*-cells [30], in the decrease of CDC25C level in different prostate cancer cell lines [9], in the differentiation of preadipocytes into adipocytes [31], and in the differentiation of human mesenchymal stem cells [32]. Finally, quinalizarin was applied as an advantageous tool to study the variation of the protein expression on one side [33] and phosphoproteome alteration [33] on another side, using HEK293T cells.

## 2. Materials and Methods

### 2.1. Inhibitors

Quinalizarin was purchased from ACP Chemicals Inc. and solved in DMSO.

### 2.2. Protein Kinases

All the recombinant *α*, *α*′, and *β* subunits of CK2 were purified as described in [34, 35]. The source of all of the other protein kinases used for selectivity profiling is described in [36].

### 2.3. *In Silico* Analysis

The crystal structures of human and* Zea Mays* CK2 were retrieved from the PDB (PDB codes: 3FL5 and 3Q9Z, 3Q9Y, 4MD7, and 3QA0) and processed in order to remove unwanted ligands and water molecules. Hydrogen atoms were added to the protein structure using standard geometries with the MOE program [37]. To minimize contacts between hydrogens, the structures were subjected to Amber99 force-field minimization until the rms (root mean square) of conjugate gradient was <0.1 kcal·mol^−1^·Å^−1^ (1 Å = 0.1 nm) keeping the heavy atoms fixed at their crystallographic positions. To strictly validate the model generated and to calibrate the docking protocol, a small database of known CK2 inhibitors was built and a set of docking runs was performed [10, 38]. After the calibration phase, quinalizarin was docked directly into the ATP-binding site of different CK2 crystal structures, by using GOLD suite [39]. Searching is conducted within a user-specified docking sphere (10 Å from the center of the binding cleft), using the genetic algorithm protocol and the GoldScore scoring function. GOLD performs a user-specified number of independent docking runs (50 in our specific case) and writes the resulting conformations and their energies in a molecular database file. Prediction of small molecule-enzyme complex stability, the quantitative analysis for nonbonded intermolecular interactions (H-bonds, transition metal, water bridges, hydrophobic and electrostatic interactions), and the RMSD (Root Mean Square Deviation) were calculated and visualized using several tools implemented in MOE suite [37]. Molecular dynamic (MD) simulations of the final complexes (parameterized with Amber99) were performed with NAMD 2.10 [40] in order to verify their stability over time; in particular 100 ns of NPT (1 atm, 300 K) MD simulation were performed after an equilibration phase of 1 ns (positional restraints were applied on carbon atoms to equilibrate the solvent around the protein).

### 2.4. Phosphorylation Assays

Native CK2 purified from rat liver and recombinant catalytic *α* subunits alone and/or in combination with *β* subunits (0.5–1 pmol) were incubated for 10 min at 37°C in a final volume of 25 *μ*L containing 50 mM Tris/HCl (pH 7.5), 100 mM NaCl, 12 mM MgCl_2_, 100 *μ*M synthetic peptide substrate RRRADDSDDDDD, and 20 *μ*M [*γ* ^33^P-ATP] (500–1000 cpm/pmol). Reaction was stopped by addition of 5 *μ*L of 0.5 M orthophosphoric acid before spotting aliquots onto phosphocellulose filters. Conditions for the activity assays of all other protein kinases tested in selectivity experiments are as described or referenced in [36].

### 2.5. Kinetic Determinations

Initial velocities were determined at each of the substrate concentrations tested. Km values were calculated either in the absence or in the presence of increasing concentrations of inhibitor, from Lineweaver-Burk double-reciprocal plots of the data. Inhibition constants were then calculated by linear regression analysis of Km/Vmax against inhibitor concentration plots.

### 2.6. Selectivity Profiles

Lorentz curves, Gini coefficients, and hit rates (expressing the percent of kinases inhibited >50% by a given compound) were calculated from the selectivity data as described in [41].

## 3. Results and Discussion

### 3.1. Quinalizarin is One of the Most Selective Inhibitors of CK2

Anthraquinones, together with flavonoids and coumarins, are one of the chemical classes of compounds most exploited as inhibitors of CK2 [10, 18, 42, 43]. Many compounds belonging to these chemical classes have been demonstrated to be potent inhibitors of protein kinase CK2; however most of them lack selectivity. Identified through a computer aided virtual screening, quinalizarin proved to be the most active anthraquinone inhibitor of CK2, with Ki value (52 nM) 3.5 order of magnitude lower than its natural analog emodin [10]. Moreover, the assay of quinalizarin against a panel of 70 protein kinases disclosed a promising selectivity, since none of the other kinases was inhibited as drastically as CK2 [10].

**Figure 1:**
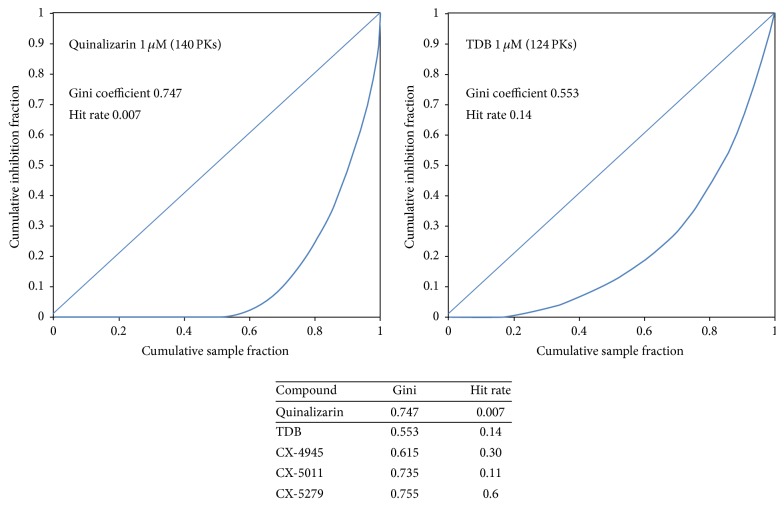
Lorenz curves, Gini coefficients, and hit rates for quinalizarin, TDB [44], CX-4945 [45], CX-5011 [45], and CX-5279 [45]. Detailed information in Section 2.

A more accurate selectivity profile of quinalizarin at a concentration of 1 *μ*M has been now performed by extending the panel to 140 protein kinases. Interestingly, the selectivity of quinalizarin appears to be even better than that inferred from the previous panel (see Table 1). In particular, CK2 holoenzyme displays a residual activity of 10%, consistent with the data previously acquired (8%, [10]). None of the other 139 protein kinases displays a residual activity less than 50%; 132 protein kinases are nearly unaffected by 1 *μ*M quinalizarin, with a residual activity equal to or more than 80%. Only seven protein kinases (PLK1, CK1*δ*, PIM3, MST2, MST4, MLK3, and BRK) exhibit a residual activity less than 80%; however the second most inhibited kinase (PIM3) still exhibits 62% residual activity. The remarkable selectivity of quinalizarin is further highlighted by drawing from the data of Table 1 the Gini coefficient (0.747) and hit rate (0.007) denoting a very specific kinase inhibitor. In particular, the Gini value is higher than those of the TDB (0.553, [44]) and of the only CK2 inhibitor in clinical trials, CX-4945 (0.615, [45]), and close to the value calculated for CX-5011 (0.735, [45]) and CX-5279 (0.755, [45]) (see Figure 1). Furthermore the hit rate of quinalizarin is the lowest ever calculated for a CK2 inhibitor, as only 0.7% of the kinase panel considered (i.e., only CK2) is inhibited more than 50% (Figure 1).

**Figure 2:**
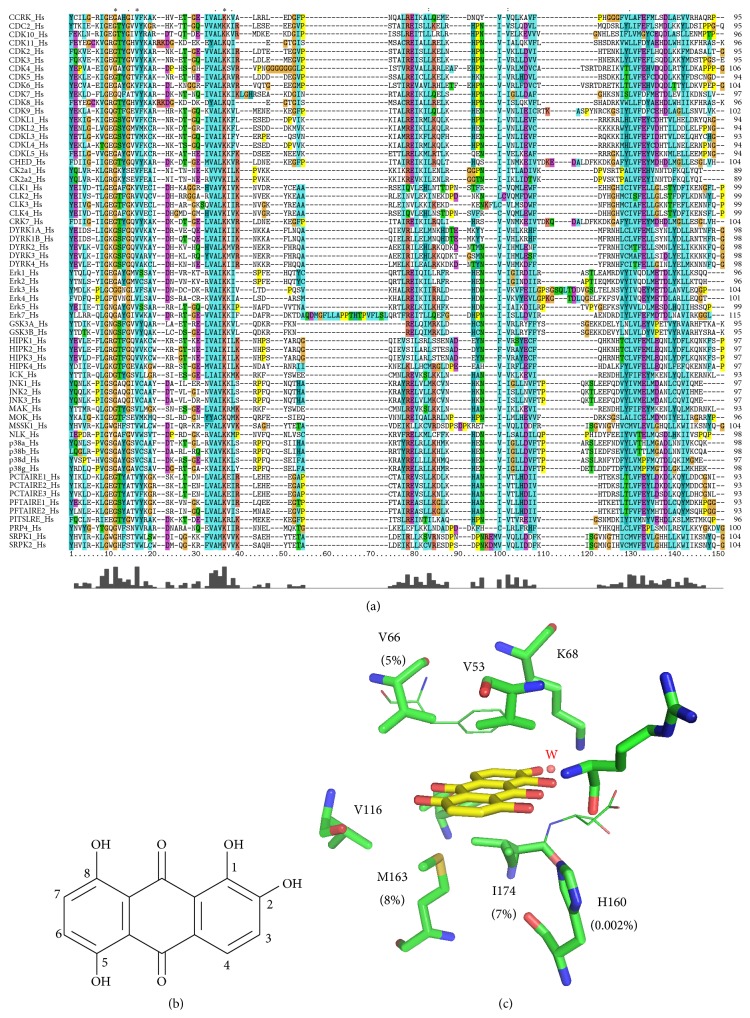
(a) Multiple alignment of the human kinome, using ClustalW 2.0. (b) Chemical structure of quinalizarin; atomic positions are highlighted. (c) Schematic representation of quinalizarin in complex with CK2*α* (PDB code: 3Q9Z); the percentage of specific residues in the kinome has been highlighted. W indicates a conserved water molecule.

To shed light on the molecular features underlying the remarkable selectivity of quinalizarin, a multiple alignment of the human kinome has been performed (Figure 2(a)), highlighting the amino acids involved in the quinalizarin binding motif, according to the crystallographic data available (PDB codes: 3FL5 [10], 3Q9Z, and 3Q9Y [29]). Quinalizarin interacts with the ATP-binding cleft by positioning close to the phosphate binding region. Crucially responsible for this interaction is the acidic hydroxyl group at position 2 (OH^2^), which is able to make a strong interaction with Lys68 and a conserved water molecule (w). Possibly, Lys68 is able to create a concentrated positive charge into the CK2 phosphodonor site, promoting the first quinalizarin deprotonation, similar to the condition occurring in mild base solution. On the other hand, different hydrophobic interactions ensure the correct positioning and direction of OH^2^ in particular, with the upper side of the cleft (Val66 and Val53) and with the bottom side (Ile174 and Met163). While Val53 is well conserved among the kinome, Val66 and Ile174 are present only in the 5% and 7% of the kinome, respectively, being generally substituted with small amino acids like alanine. Met163 position, on the contrary, is generally occupied by bulkier residues like Leu, Ile, and Phe and is found as such only in 8% of the kinome. To sum up, the coexistence of all these hydrophobic residues inside the ATP-binding cleft is very rare in the human kinome. Moreover, two other hydrogen bonds contribute to the quinalizarin binding motif: the first one between the hydroxyl group in position 5 (OH^5^) and the carbonyl backbone of Val116 in the hinge region via a water molecule; the other one is between the hydroxyl group in position 8 (OH^8^) and, on one side, His160, and, on the other side, the backbone carbonyl group of Arg47 from p-loop. Both* Zea maysα* (pH 7.5) and human *α* (pH 6.5) complexes with quinalizarin present this particular interaction between (OH^8^), His160 (conformation “up”), and Arg47, stabilizing p-loop in a close conformation, a unique situation among all the other CK2 crystal structures. On the contrary, the quinalizarin/human CK2*α* complex at pH 8.5 (PDB code: 3Q9Y) presents the canonical conformation in which p-loop and His160 do not interact, adopting the common p-loop “open conformation,” His160 “down.” This pH condition, however, is far away from both the physiological conditions and the experimental conditions adopted* in vitro*. Anyway, no other protein kinase presents a histidine at position 160; this feature in conjunction with the unique amino acidic distributions in the binding cleft supports the conclusion that quinalizarin binding motif is by itself responsible for the outstanding selectivity of this inhibitor (Figure 2(b)).

### 3.2. Quinalizarin Differentiates between CK2 Alpha and Tetramer

**Figure 3:**
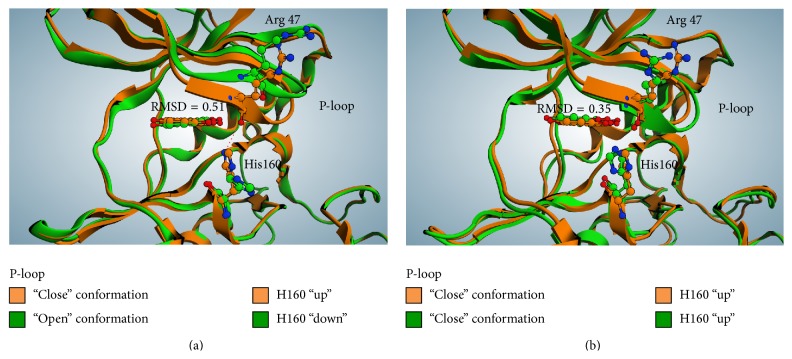
(a) Superimposition of quinalizarin/CK2*α* complex (PDB code: 3Q9Z, orange) and quinalizarin/CK2*α* docking complex, from CK2*α* apo form (PDB code: 3QA0, green); (b) superimposition of the described structures after 100 ns of molecular dynamic simulation. Details about p-loop and His160 conformations are highlighted.

**Figure 4:**
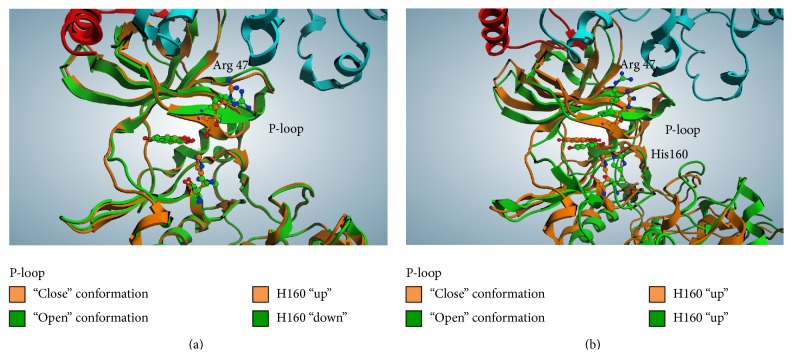
(a) Superimposition of quinalizarin/CK2*α* complex (PDB code: 3Q9Z, orange) and quinalizarin/CK2 tetramer docking complex, from CK2 tetrameric apo form (PDB code: 4MD7, green); (b) superimposition of the described structures after 100 ns of molecular dynamic simulation. Details about p-loop and His160 conformations are highlighted.

By looking at Table 1, a clear difference between the residual activity of CK2 alpha and CK2 tetramer can be observed. In fact the low residual activity value of CK2 tetramer (10%) is replaced by an unexpected high value in the case of CK2 alpha alone (42%). To confirm these data IC_50_ and Ki values of quinalizarin with respect to CK2 holoenzyme and CK2*α* alone have been determined (Table 2). IC_50_ value of quinalizarin for CK2 holoenzyme (0.15 *μ*M, close to the value previously published [10]) is one order of magnitude lower than the value calculated for CK2*α* alone (IC_50_ = 1.35 *μ*M) and consistent with the residual activity disclosed in the kinase panel (Table 1). Likewise also Ki values are different, 0.058 *μ*M and 0.675 *μ*M, respectively (see Table 2). Even though the mechanism of action of quinalizarin is ATP competitive in both cases ([10] and data not shown), the results clearly demonstrate that quinalizarin is more effective against CK2 tetramer as compared to CK2*α*. To extend this information the residual activity of other CK2 forms has been evaluated at 1 *μ*M concentration of quinalizarin (Table 3). The recombinant human CK2*α*′ denotes a residual activity (38%) nearly identical to the one calculated for CK2*α* (42%) and* Zea mays* CK2*α* (33%); as expected the residual activity drops to 14% in the case of the recombinant tetramer CK2*α*_2_′*β*_2_. Interestingly, also the native (nCK2) tetrameric enzyme purified from rat liver displays a negligible residual activity (6%) when treated with 1 *μ*M quinalizarin consistent with the concept that in these native preparations by far predominant form of CK2 is the holoenzyme, while the isolated catalytic subunits must be nearly absent. To try to understand the molecular features underlying the different inhibitory efficiency of quinalizarin against CK2 tetramer with respect to CK2*α*, a two-step computational study has been performed. Firstly a docking simulation was performed using CK2 holoenzyme crystal structure apo form (PDB code: 4MD7, [46]) and CK2*α* apo form (PDB code: 3QA0, [29]) and compared to the crystallographic pose of quinalizarin (PDB code: 3Q9Z). The docking and the crystallographic poses were nearly superimposable (RMSD = 0.35 Å and 0.51 Å, resp.; see Figures 3(a) and 4(a)), to note that both apo crystal structures present an “open” conformation of p-loop/His160 (Figures 3(a) and 4(a)), while the quinalizarin/CK2*α* complexes are in the “close” one (see Section 3.1). Secondly a molecular dynamic simulation was performed on both docking complexes to study their conformations over time. After 100 ns of dynamics simulation the quinalizarin/CK2*α* docking complex displays a very similar conformation as compared to quinalizarin/human CK2*α* crystal structure. In particular, as shown in Figure 3(b), p-loop conformation dramatically changes from the starting “open” condition to the “close” one identified in the human and* Zea mays* crystal structures (PDB codes: 3FL5 and 3Q9Z). On the other hand, His160 restores the interaction with both quinalizarin OH^8^ and the backbone carboxyl group of Arg47 (Figure 3(b)). The distance calculated between His160 and quinalizarin OH^8^ is 3.02 Å and towards the carboxyl group of Arg47 it is 2.98 Å. These values are close to the ones exhibited in the quinalizarin/human CK2*α* crystallographic structure (3.19 Å and 3.21 Å, resp.). In other words, the molecular dynamics simulation was able to reproduce the crystal structure conformation of quinalizarin/human CK2*α*, starting from a completely unrelated CK2*α* apo form; this result strengthens the idea that CK2*α* conformation identified in complex with quinalizarin is due to the presence of the inhibitor inside the ATP pocket.

**Figure 5:**
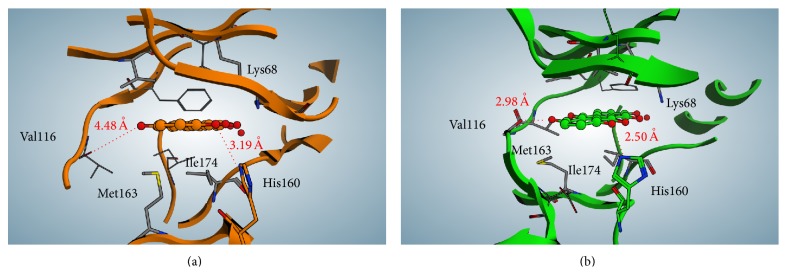
Comparison between the binding motif of quinalizarin inside CK2*α* crystallographic structure (PDB code: 3Q9Z, orange) and CK2 tetramer (PDB code: 4MD7, green) after 100 ns of molecular dynamic simulation.

On the contrary, the molecular dynamic simulation of the quinalizarin/CK2 tetramer complex highlights some differences in quinalizarin binding motif as compared to the one observed in the case of CK2*α* alone. First of all, p-loop conformation remains in an “open” state (Figures 4(a) and 4(b)); this condition is probably due to the interactions between the two beta subunits and a few residues in p-loop, namely, Arg47, Lys49, Lys44, Glu52, and Phe54. On the other side, His160 assumes the “up” conformation, interacting directly with quinalizarin OH^8^, without the interference of the carboxyl group of Arg47 of p-loop (Figure 4(b)). Secondly, by comparing the crystal structure of the quinalizarin/human CK2 complex (PDB: 3Q9Z) and the quinalizarin/human CK2 tetrameric complex obtained from the docking/molecular dynamics techniques, we can see that several amino acids of the binding site are differently organized around quinalizarin (Figures 5(a) and 5(b)). In fact, while in the case of quinalizarin/CK2 crystallographic complex the p-loop assumes the “close” conformation, in the case of quinalizarin/CK2 tetramer model, the p-loop is sitting in a planar conformation with respect to the inhibitor, thus reinforcing the hydrophobic interactions between the inhibitor and the binding site. Moreover, while the interaction between Lys68 and the quinalizarin OH^2^ is conserved in both cases either in terms of distances or in terms of directions, His160 interacts more efficiently with quinalizarin OH^8^ in the quinalizarin/CK2 tetramer model compared to the crystallographic complex. In fact, the distance between His160 and quinalizarin OH^8^ retrieved from the crystallographic complex 3Q9Z (3.19 Å) is drastically reduced to 2.5 Å in the case of quinalizarin/CK2 tetramer model (Figures 5(a) and 5(b)). On the other side, the hinge region of the quinalizarin/tetrameric complex is arranged 1.5 Å closer to the inhibitor, thus allowing a direct hydrogen bond between the carboxyl group of Val116 and quinalizarin (OH^5^).

In conclusion, the data presented provide the clear-cut demonstration that quinalizarin is one of most selective inhibitors of protein kinase CK2, with a high Gini coefficient and the lowest hit rate ever reported. Moreover the ability of quinalizarin to discriminate between CK2*α* and CK2*α*_2_ *β*_2_, being more effective against CK2 holoenzyme, has been disclosed both by* in vitro* experiments and by* in silico* analysis. Given that quinalizarin is cell permeable, the new information provided in this paper will be relevant also to cell studies affording a tool for the estimation of different CK2 forms in the cell and to the identifications of substrates specifically targeted by either CK2 holoenzyme or its isolated catalytic subunits.

## Figures and Tables

**Table 1:** Selectivity profiles of quinalizarin on a 140-kinase panel. Residual CK2 activity (determined at 1 *μ*M quinalizarin concentration) is expressed as a percentage of the control activity without inhibitor. Conditions are described or referenced in the experimental section. Activities <50% of control are bold typed.

Kinase	Activity %	Kinase	Activity %	Kinase	Activity %
**C** **K**2**α**_2_ **β**_2_	**10**	PKCa	**95**	TAK1	**107**
**C** **K**2**α**	**42**	IRAK4	**95**	CDK9-Cyclin T1	**108**
PIM3	**62**	HIPK3	**96**	RSK1	**108**
MLK3	**63**	JNK3	**96**	MPSK1	**108**
CK1*δ*	**72**	IGF-1R	**96**	RSK2	**108**
BRK	**72**	VEG-FR	**96**	DDR2	**109**
PLK1	**75**	IRAK1	**97**	EPH-A2	**110**
MST2	**77**	MAPKAP-K2	**97**	MNK1	**110**
MST4	**78**	PAK5	**97**	PKC*γ*	**110**
CHK2	**80**	IKKe	**97**	CHK1	**111**
MKK1	**81**	GSK3b	**98**	OSR1	**111**
TrkA	**81**	DYRK1A	**98**	JNK2	**112**
PKBb	**81**	MAPKAP-K3	**98**	STK33	**112**
CAMK1	**82**	MSK1	**99**	NEK6	**112**
MKK2	**82**	AMPK (hum)	**99**	IKKb	**112**
ABL	**82**	SYK	**99**	EIF2AK3	**112**
PDK1	**83**	PDGFRA	**99**	p38g MAPK	**114**
MAP4K3	**84**	LKB1	**99**	MKK6	**114**
MLK1	**84**	p38a MAPK	**99**	ZAP70	**114**
PIM1	**85**	HIPK2	**99**	p38b MAPK	**114**
FGF-R1	**85**	HER4	**100**	CSK	**114**
MAP4K5	**86**	MARK2	**100**	TTBK1	**115**
PAK6	**87**	TTBK2	**100**	Aurora A	**115**
TIE2	**88**	EPH-B2	**101**	TAO1	**115**
MNK2	**88**	TTK	**101**	MEKK1	**115**
SIK2	**89**	ULK2	**101**	MELK	**116**
MARK3	**89**	WNK1	**101**	SRPK1	**117**
YES1	**89**	ERK8	**102**	EPH-B3	**117**
GCK	**90**	PINK	**102**	PRK2	**118**
ERK1	**90**	PKCz	**102**	PIM2	**120**
TESK1	**90**	PAK4	**102**	IRR	**120**
PKBa	**91**	JAK2	**102**	ASK1	**120**
DYRK2	**91**	MARK4	**103**	p38d MAPK	**120**
SGK1	**91**	BRSK1	**103**	PKA	**121**
CK1*γ*2	**91**	PRAK	**103**	CDK2-Cyclin A	**121**
S6K1	**91**	RIPK2	**103**	Lck	**122**
CLK2	**91**	TBK1	**103**	HIPK1	**123**
SmMLCK	**92**	DYRK3	**104**	BTK	**125**
JNK1	**92**	NUAK1	**104**	EPH-A4	**126**
ULK1	**92**	NEK2a	**105**	TLK1	**126**
Aurora B	**93**	SIK3	**105**	MST3	**126**
DAPK1	**93**	ROCK 2	**105**	IR	**128**
ERK2	**94**	MINK1	**105**	TGFBR1	**130**
CAMKKb	**94**	ERK5	**106**	TSSK1	**131**
EF2K	**94**	EPH-B1	**106**	EPH-B4	**132**
BRSK2	**95**	PAK2	**106**		
PKD1	**95**	MARK1	**106**		
Src	**95**	PHK	**107**		

**Table 2:** IC_50_ and Ki values of quinalizarin for CK2*α*_2_ *β*_2_ and CK2*α*.

	CK2*α*_2_ *β*_2_	CK2*α*
IC_50_ (*μ*M)	0.15 ± 0.02	1.35 ± 0.15
Ki (*μ*M)	0.058 ± 0.003	0.675 ± 0.19

**Table 3:** Residual catalytic activity (determined at 1 *μ*M quinalizarin concentration) of different CK2 forms.

CK2 form	Activity %
CK2*α* (Human)	42%
CK2*α*′ (Human)	38%
CK2*α* (Zea mays)	33%

CK2*α*_2_ *β*_2_ (Human)	10%
CK2*α*_2_′*β*_2_ (Human)	14%
nCK2 (Rat liver)	6%
